# Supplementary material for: Multi-ancestry GWAS of age-related hearing loss identifies 140 loci and key cellular mechanisms
Source: Nat Commun. 2026 Feb 21;17:4325. doi: 10.1038/s41467-026-69894-z (PMC13172361; doi:10.1038/s41467-026-69894-z)
Supplement: Supplementary file 1 — Supplementary Information [file 41467_2026_69894_MOESM1_ESM.pdf]

Supplementary Information

Multi-ancestry GWAS of age-related hearing loss identifies 140 loci and key cellular mechanisms

Shi *et al.*

Contents

Supplementary Figure 1-15

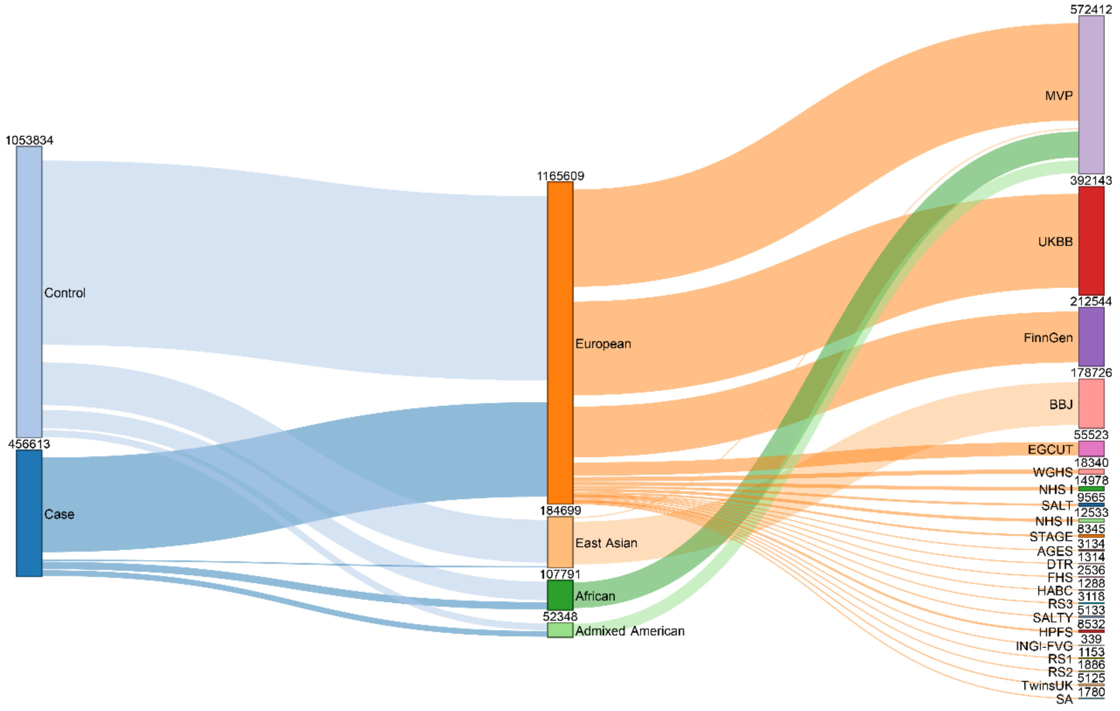

Supplementary Figure 1 Overview of GWAS summary statistics used in the cross-ancestry meta-analysis of ARHL. Abbreviations: MVP, Million Veteran Program; UKBB, UK Biobank; BBJ, Biobank Japan; EGCUT, Estonian Genome Center at the University of Tartu; WGHS, Women's Genome Health Study; SALT, Screening Across the Lifespan Twin; RS, Rotterdam Study; STAGE, Screening Twin Adults: Genes and Environment; AGES, Age, Genes/Environment Susceptibility – Reykjavik; DTR, Danish Twin Registry; FHS, Framingham Heart Study; SALTY, Screening Across the Lifespan Twin young; HABC, Health ABC – EUR; INGI-FVG, Italian Network of Genetic Isolates - Friuli Venezia Giulia; SA, the Salus in Apulia study; NHS, Nurses' Health Studies; HPFS, Health Professionals Follow-up Study.

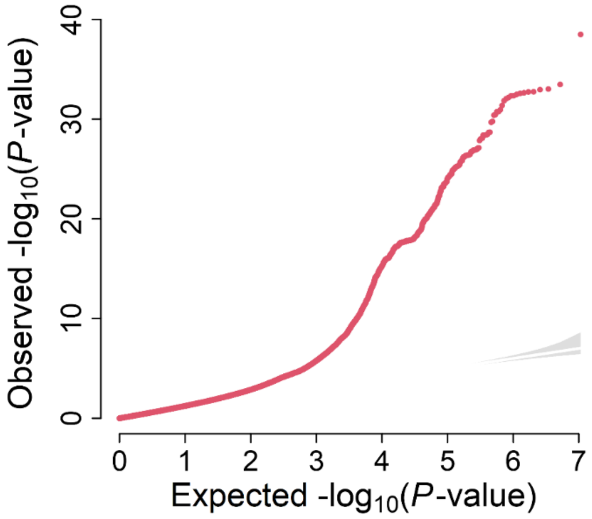

Supplementary Figure 2 Quantile-quantile (QQ) plot of cross-ancestry GWAS meta-analysis of ARHL. The QQ plot shows the observed versus expected $-\log_{10}(p)$ under the null hypothesis of no association. Deviation from the diagonal line is primarily due to polygenicity rather than confounding factors such as population stratification (**Methods**).

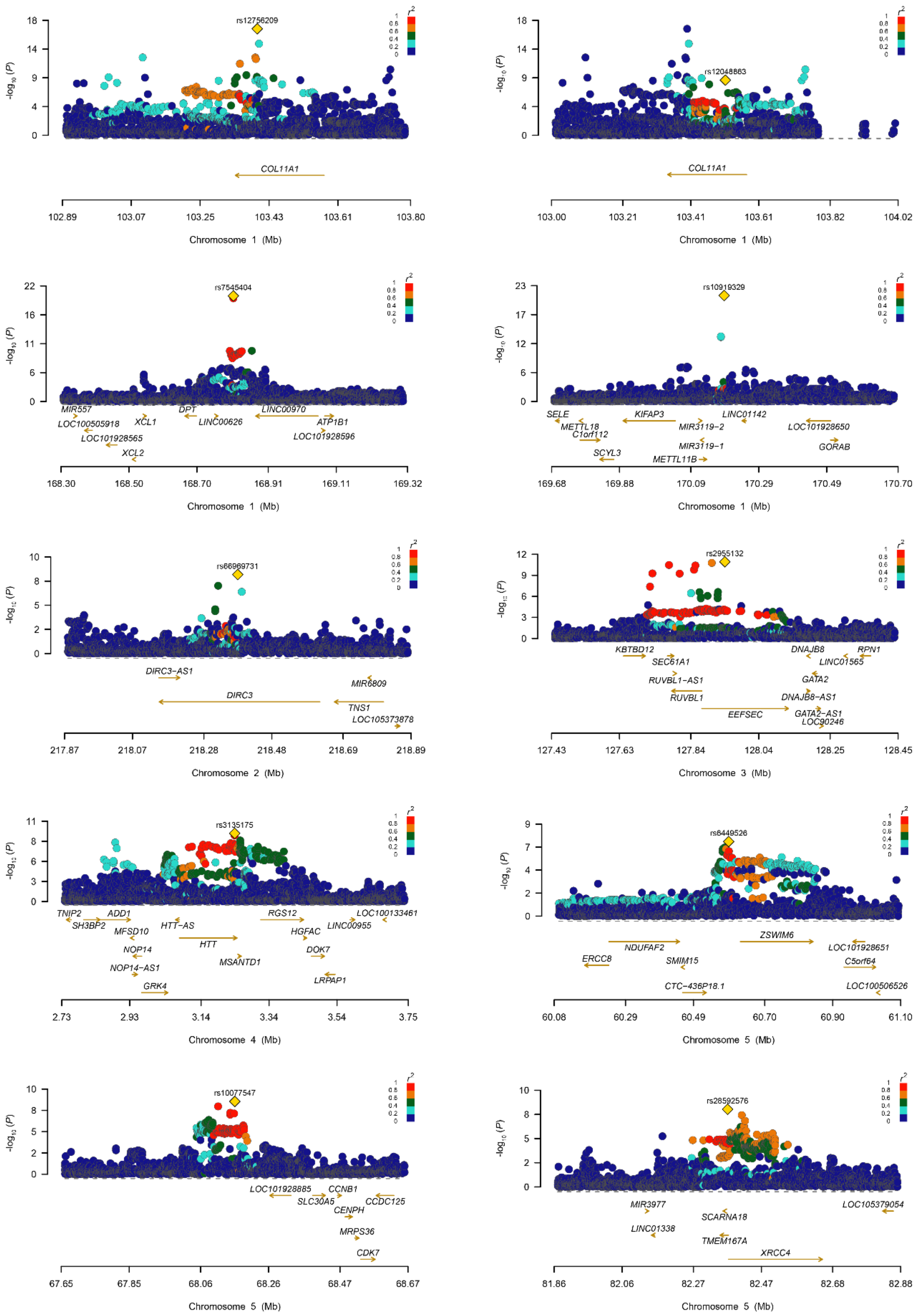

rs12756209
COL11A1
Chromosome 1 (Mb)
rs12048863
COL11A1
Chromosome 1 (Mb)
rs7545404
MIR557
LOC100505918
LOC101928565
XCL1
XCL2
DPT
LINC00626
LINC00970
ATP1B1
LOC101928596
Chromosome 1 (Mb)
rs10919329
SELE
METTL18
C1orf112
SCYL3
KIFAP3
MIR3119-2
MIR3119-1
METTL11B
LINC01142
LOC101928650
GORAB
Chromosome 1 (Mb)
rs66969731
DIRC3-AS1
DIRC3
MIR6809
TNS1
LOC105373878
Chromosome 2 (Mb)
rs2955132
KBTBD12
SEC61A1
RUVBL1-AS1
RUVBL1
EEFSEC
DNAJB8
RPN1
LINC01565
GATA2
DNAJB8-AS1
GATA2-AS1
LOC90246
Chromosome 3 (Mb)
rs3135175
TNIP2
ADD1
SH3BP2
MFSD10
NOP14
NOP14-AS1
GRK4
HTT-AS
HTT
MSANTD1
RGS12
HGFAC
LINC00955
LOC100133461
DOK7
LRPAP1
Chromosome 4 (Mb)
rs6449526
NDUFAF2
ERCC8
SMIM15
CTC-436P18.1
ZSWIM6
LOC101928651
C5orf64
LOC100506526
Chromosome 5 (Mb)
rs10077547
LOC101928885
CCNB1
SLC30A5
CCDC125
CENPH
MRPS36
CDK7
Chromosome 5 (Mb)
rs28592576
MIR3977
LINC01338
SCARNA18
TMEM167A
XRCC4
LOC105379054
Chromosome 5 (Mb)

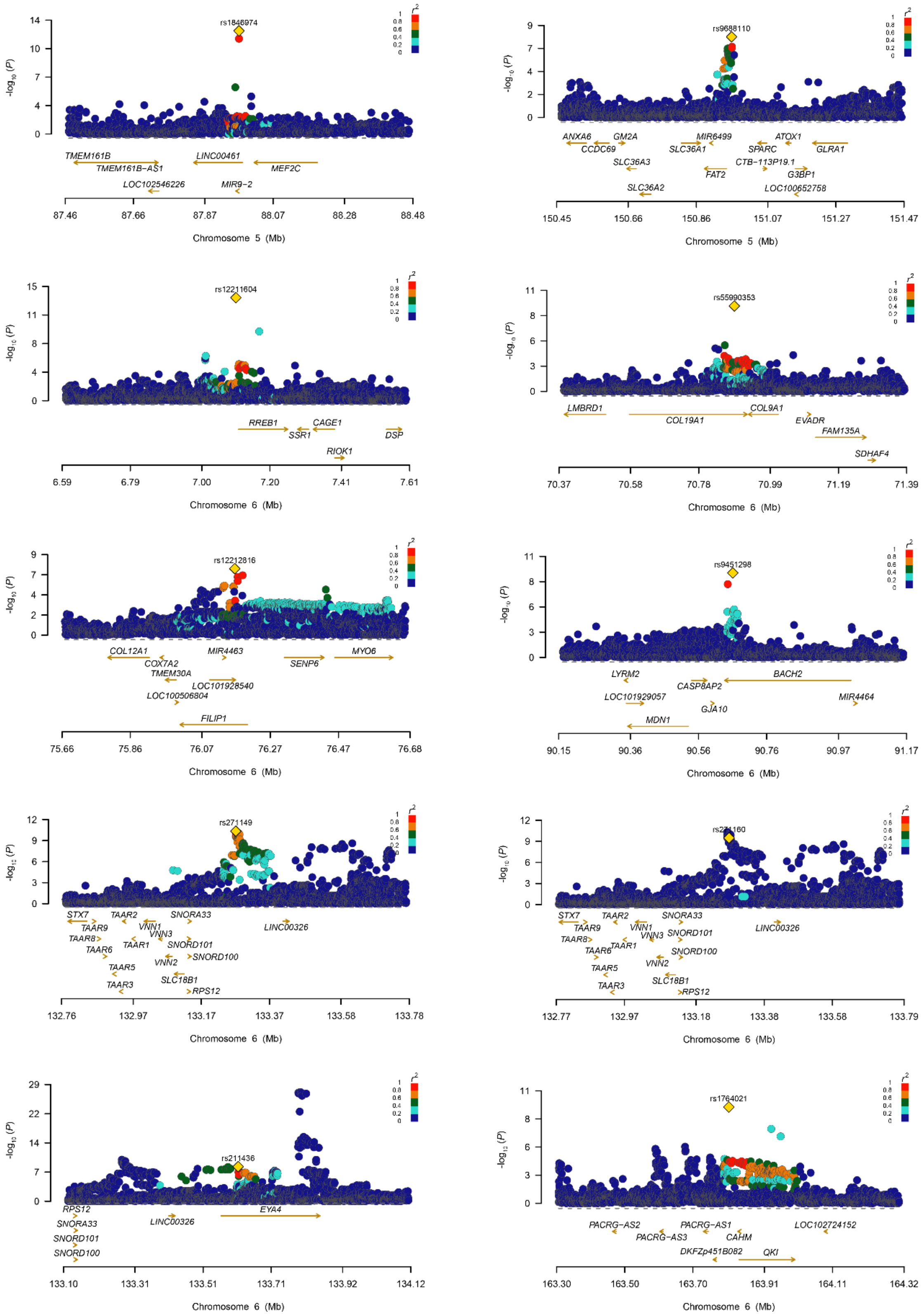
rs1846974
TMEM161B
TMEM161B−AS1
LOC102546226
LINC00461
MIR9−2
MEF2C
Chromosome 5 (Mb)
rs9688110
ANXA6
CCDC69
GM2A
SLC36A3
SLC36A2
MIR6499
SLC36A1
FAT2
SPARC
ATOX1
CTB−113P19.1
G3BP1
GLRA1
LOC100652758
Chromosome 5 (Mb)
rs12211604
RREB1
SSR1
CAGE1
RIOK1
DSP
Chromosome 6 (Mb)
rs55990353
LMBRD1
COL19A1
COL9A1
EVADR
FAM135A
SDHAF4
Chromosome 6 (Mb)
rs12212816
COL12A1
COX7A2
TMEM30A
LOC100506804
MIR4463
LOC101928540
FILIP1
SENP6
MYO6
Chromosome 6 (Mb)
rs9451298
LYRM2
LOC101929057
MDN1
CASP8AP2
GJA10
BACH2
MIR4464
Chromosome 6 (Mb)
rs271149
STX7
TAAR9
TAAR8
TAAR6
TAAR5
TAAR3
TAAR2
TAAR1
VNN1
VNN3
VNN2
SLC18B1
SNORA33
SNORD101
SNORD100
RPS12
LINC00326
Chromosome 6 (Mb)
LINC00326
Chromosome 6 (Mb)
rs211436
RPS12
SNORA33
SNORD101
SNORD100
LINC00326
EYA4
Chromosome 6 (Mb)
rs1764021
PACRG−AS2
PACRG−AS3
PACRG−AS1
CAHM
DKFZp451B082
QKI
LOC102724152
Chromosome 6 (Mb)

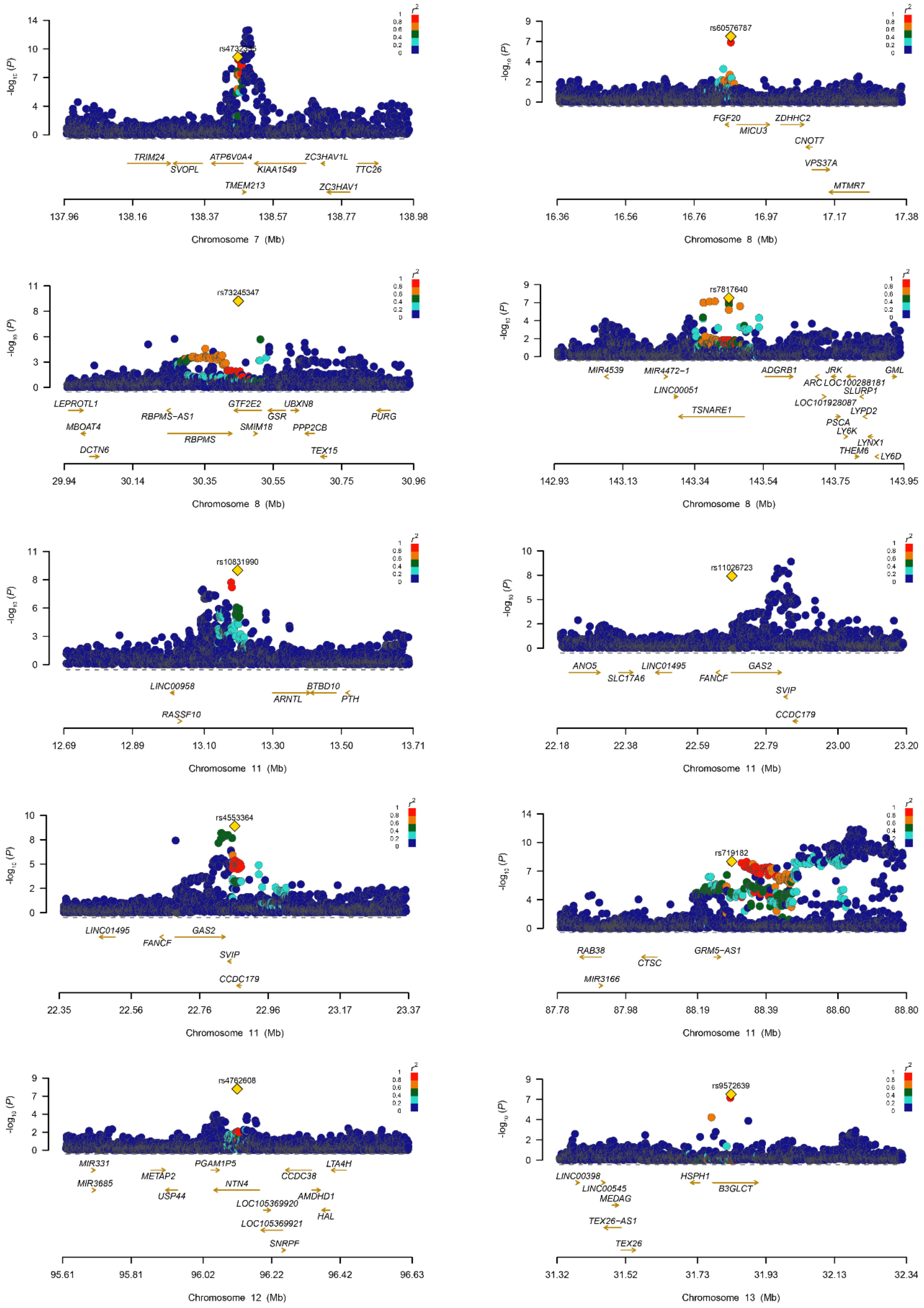

rs60576787
FGF20
MICU3
ZDHHC2
CNOT7
VPS37A
MTMR7
Chromosome 8 (Mb)
TRIM24
SVOPL
ATP6V0A4
KIAA1549
ZC3HAV1L
TTC26
TMEM213
ZC3HAV1
Chromosome 7 (Mb)
rs73245347
LEPROTL1
RBPMS-AS1
GTF2E2
GSR
UBXN8
PURG
MBOAT4
SMIM18
PPP2CB
RBPMS
DCTN6
TEX15
Chromosome 8 (Mb)
rs7817640
MIR4539
MIR4472-1
ADGRB1
JRK
GML
ARC
LOC100288181
SLURP1
LINC00051
LOC101928087
LYPD2
TSNARE1
PSCA
LY6K
LYNX1
THEM6
LY6D
Chromosome 8 (Mb)
rs10831990
LINC00958
BTBD10
ARNTL
PTH
RASSF10
Chromosome 11 (Mb)
rs11026723
ANO5
LINC01495
GAS2
SLC17A6
FANCF
SVIP
CCDC179
Chromosome 11 (Mb)
rs4553364
LINC01495
GAS2
FANCF
SVIP
CCDC179
Chromosome 11 (Mb)
rs719182
RAB38
GRM5-AS1
CTSC
MIR3166
Chromosome 11 (Mb)
rs4762608
MIR331
PGAM1P5
LTA4H
METAP2
CCDC38
MIR3685
NTN4
USP44
AMDHD1
LOC105369920
HAL
LOC105369921
SNRPF
Chromosome 12 (Mb)
rs9572639
LINC00398
HSPH1
LINC00545
B3GLCT
MEDAG
TEX26-AS1
TEX26
Chromosome 13 (Mb)

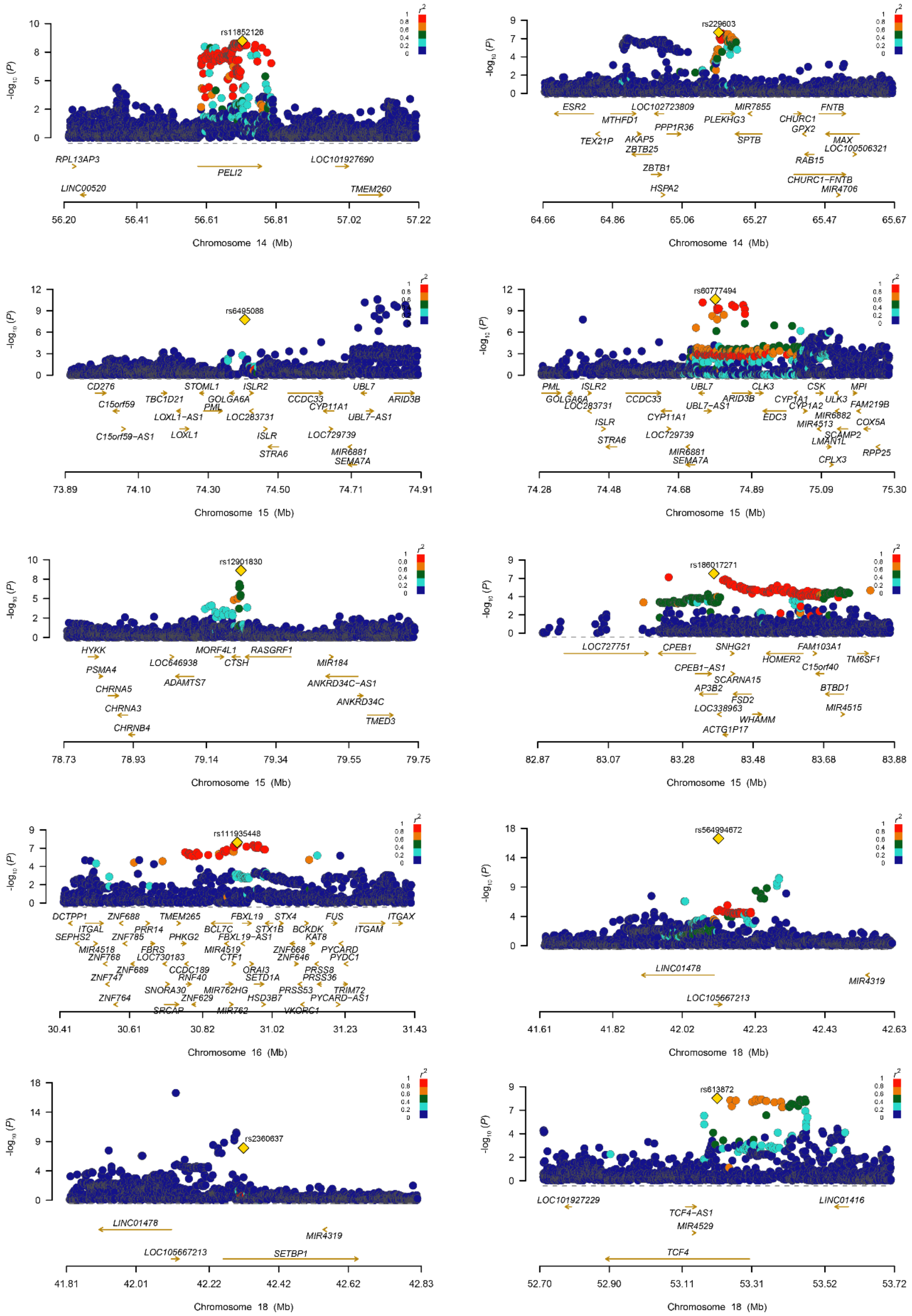

rs11852126
RPL13AP3
PELI2
LOC101927690
LINC00520
TMEM260
56.20 56.41 56.61 56.81 57.02 57.22
Chromosome 14 (Mb)
rs229603
ESR2
LOC102723809
MIR7855
FNTB
MTHFD1
PLEKHG3
CHURC1
PPP1R36
GPX2
TEX21P
AKAP5
SPTB
MAX
ZBTB25
LOC100506321
RAB15
ZBTB1
CHURC1-FNTB
HSPA2
MIR4706
64.66 64.86 65.06 65.27 65.47 65.67
Chromosome 14 (Mb)
rs6495088
CD276
STOML1
ISLR2
UBL7
C15orf59
TBC1D21
GOLGA6A
CCDC33
ARID3B
PML
CYP11A1
LOXL1-AS1
LOC283731
UBL7-AS1
C15orf59-AS1
LOXL1
ISLR
LOC729739
STRA6
MIR6881
SEMA7A
73.89 74.10 74.30 74.50 74.71 74.91
Chromosome 15 (Mb)
rs60777494
PML
ISLR2
UBL7
CLK3
CSK
MPI
GOLGA6A
CCDC33
ARID3B
CYP1A1
ULK3
LOC283731
UBL7-AS1
CYP1A2
FAM219B
CYP11A1
EDC3
MIR6882
COX5A
ISLR
MIR4513
SCAMP2
STRA6
LOC729739
LMAN1L
RPP25
MIR6881
SEMA7A
CPLX3
74.28 74.48 74.68 74.89 75.09 75.30
Chromosome 15 (Mb)
rs12901830
HYKK
MORF4L1
RASGRF1
LOC646938
CTSH
MIR184
PSMA4
ADAMTS7
ANKRD34C-AS1
CHRNA5
ANKRD34C
CHRNA3
TMED3
CHRNB4
78.73 78.93 79.14 79.34 79.55 79.75
Chromosome 15 (Mb)
rs186017271
LOC727751
CPEB1
SNHG21
FAM103A1
HOMER2
TM6SF1
CPEB1-AS1
C15orf40
SCARNA15
AP3B2
BTBD1
FSD2
LOC338963
MIR4515
WHAMM
ACTG1P17
82.87 83.07 83.28 83.48 83.68 83.88
Chromosome 15 (Mb)
rs111935448
DCTPP1
ZNF688
TMEM265
FBXL19
STX4
FUS
ITGAX
ITGAL
PRR14
BCL7C
STX1B
BCKDK
ITGAM
SEPHS2
ZNF785
PHKG2
FBXL19-AS1
KAT8
MIR4518
FBRS
MIR4519
ZNF668
PYCARD
ZNF768
LOC730183
CTF1
ZNF646
PYDC1
ZNF689
CCDC189
ORAI3
PRSS8
ZNF747
RNF40
SETD1A
PRSS36
SNORA30
MIR762HG
PRSS53
TRIM72
ZNF764
ZNF629
HSD3B7
PYCARD-AS1
SRCAP
MIR762
VKORC1
30.41 30.61 30.82 31.02 31.23 31.43
Chromosome 16 (Mb)
rs564994672
LINC01478
MIR4319
LOC105667213
41.61 41.82 42.02 42.23 42.43 42.63
Chromosome 18 (Mb)
rs2360637
LINC01478
MIR4319
LOC105667213
SETBP1
41.81 42.01 42.22 42.42 42.62 42.83
Chromosome 18 (Mb)
rs613872
LOC101927229
LINC01416
TCF4-AS1
MIR4529
TCF4
52.70 52.90 53.11 53.31 53.52 53.72
Chromosome 18 (Mb)
-log10 (P)
r2

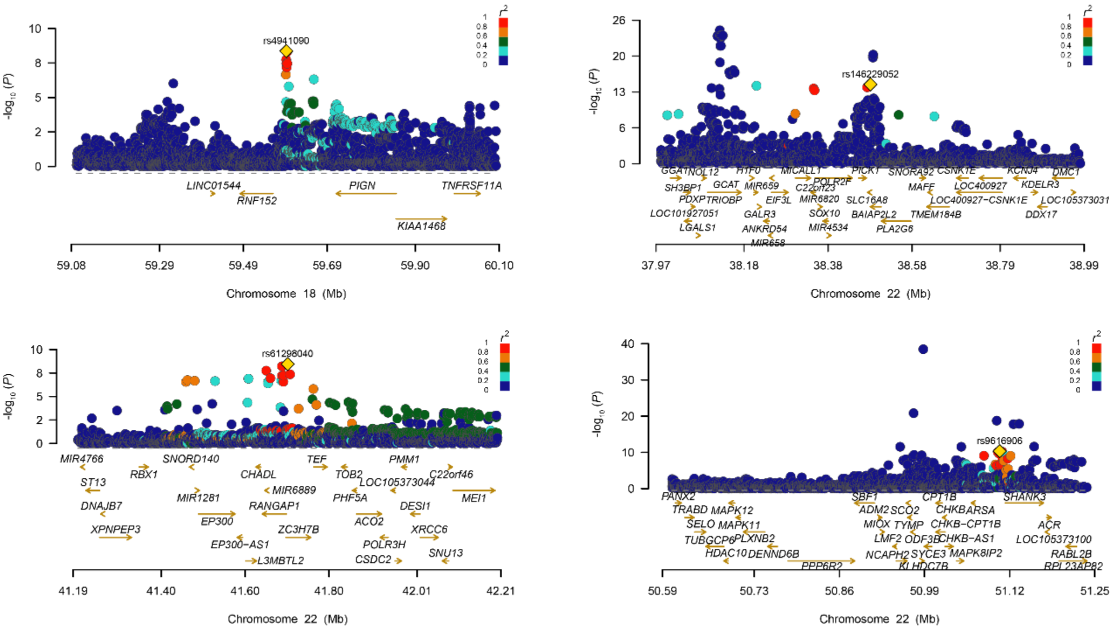

Supplementary Figure 3 Regional association plots of the 44 novel loci identified from the cross-ancestry meta-analysis at *P* < 5×10^{-8}. Each point represents a SNP passing quality control in the meta-analysis, with its *P* value (on a -log$_{10}$ scale) plotted against its genomic position (hg 19). In each plot, the lead SNP at each locus is highlighted as a gold rhombus. Other SNPs are colored according to their LD with the lead SNP (estimated from 1000 Genomes project phase 3).

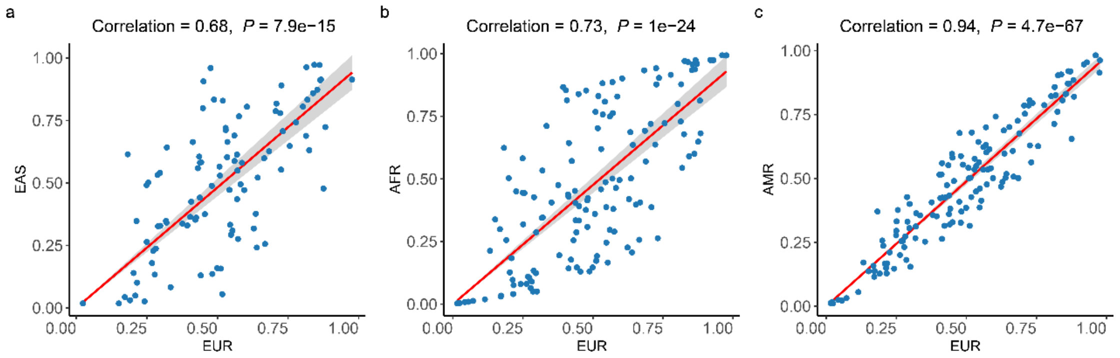

Supplementary Figure 4 Pairwise comparisons of lead SNP effect allele frequency between European and three other ancestry groups, East Asian (a), African (b) and Admixed American (c). Each dot shows the effect size of a lead SNP, and the error bars show the 95% confidence interval of the estimated effect sizes. Abbreviations: EUR, European ancestry; EAS, East Asian ancestry; AMR, Admixed American ancestry; AFR, African ancestry.

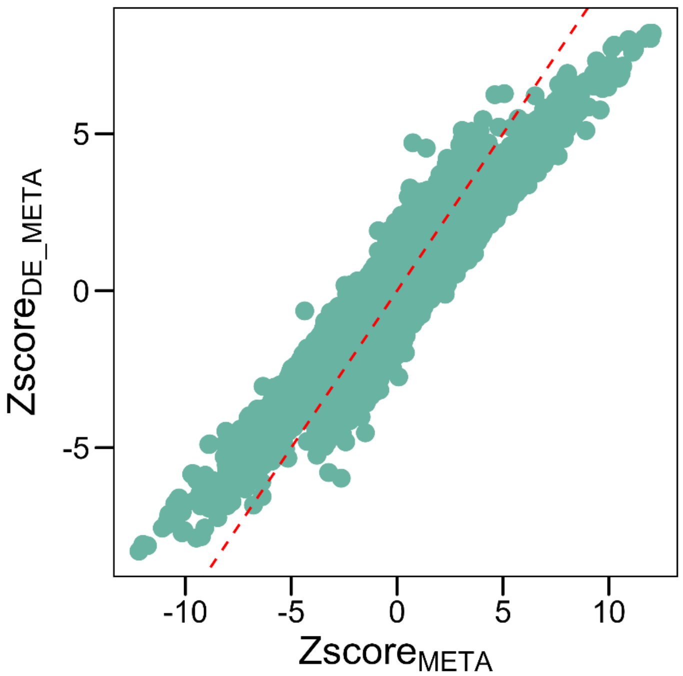

Supplementary Figure 5 Effect size comparison between the GWAS meta-analysis and the de-meta-analysis. Each point represents a genetic variant's effect size estimate from both analyses. The red dashed line indicated the identity line (i.e., $y = x$). META: the cross-ancestry meta-analysis, DE_META: the de-meta-analysis to remove the UK Biobank derived signals from the cross-ancestry meta-analysis.

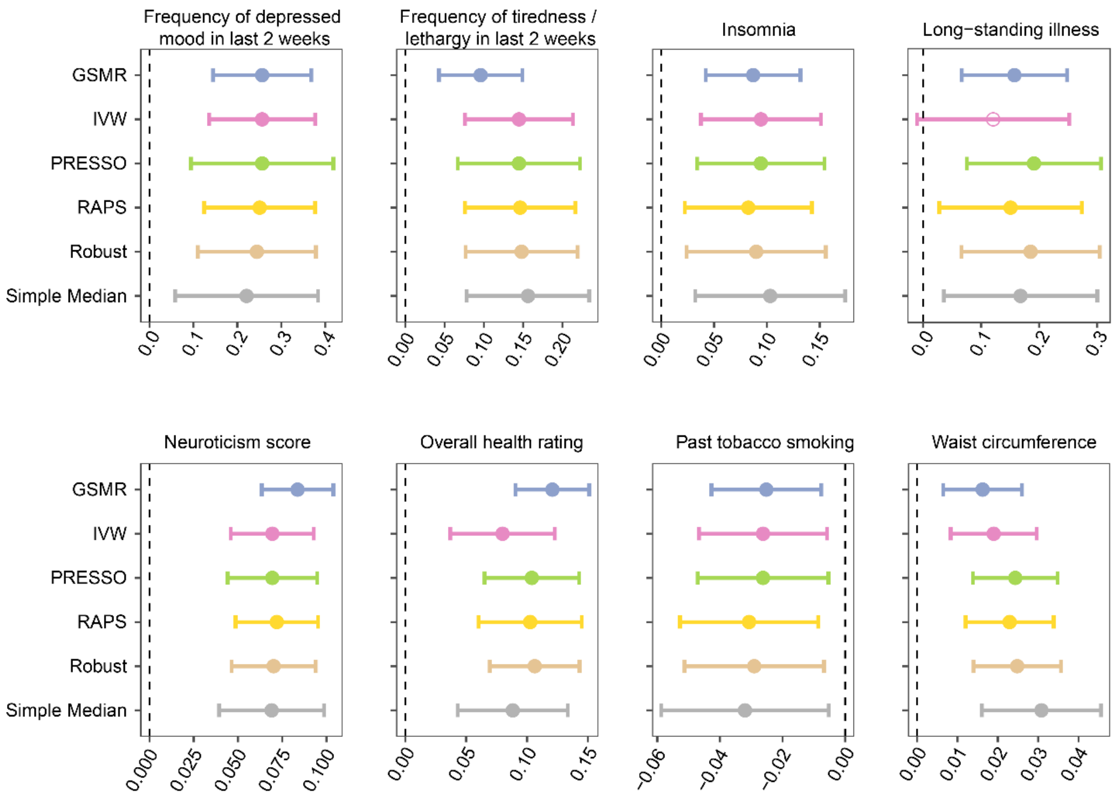

Supplementary Figure 6 Causal effect estimates from multiple Mendelian Randomization (MR) methods using ARHL as outcome. Each panel represents one exposure trait; the y-axis shows the MR methods used, and the x-axis shows the corresponding effect estimates for ARHL as the outcome. Error bars indicate 95% CI computed as $\hat{b}_{xy}$ ± 1.96 × se, where se is the standard error estimated from the multiple MR methods. Statistical significance (P < 0.05) is indicated by a solid circle.

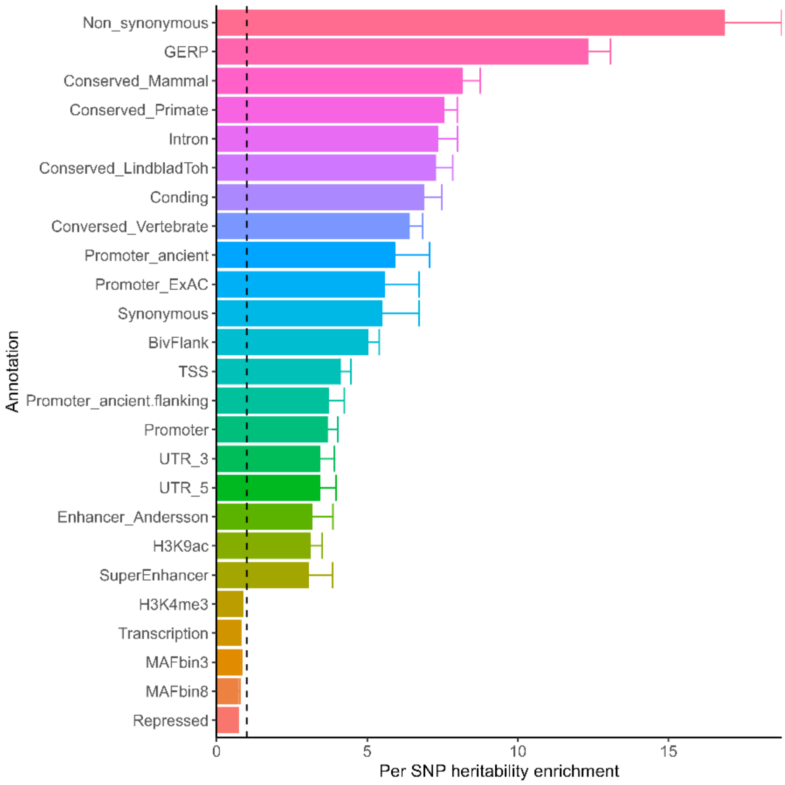

Supplementary Figure 7 Per-SNP heritability enrichment for the top 20 and bottom 5 functional categories. This figure shows the top 20 and bottom 5 functional categories ranked by per-SNP heritability enrichment using SBayesRC. Functional categories correspond to annotations from the BaseLineLD model v2.2. Error bars denote the posterior standard deviation (posterior SD) obtained from the Markov Chain Monte Carlo (MCMC) samples of the SBayeRC model. GERP, GERP.RSsup4; Conserved_primate, Conserved_Primate_phastCons46way; Intron, Intron_UCSC.flanking.500; Conserved_Mammal, Conserved_Mammal_phastCons46way; Promoter_ancient, Ancient_Sequence_Age_Human_Promoter; Conding, Coding_UCSC; Conversed_Vertebrate, Conserved_Vertebrate_phastCons46way; Promoter_ExAC, Human_Promoter_Villar_ExAC; UTR_3, UTR_3_UCSC; TSS, TSS_Hoffman; UTR_5, UTR_5_UCSC; H3K9ac, H3K9ac_peaks_Trynka; Enhancer_ancient, Ancient_Sequence_Age_Human_Enhancer; Promoter, Human_Promoter_Villar; H3K4me3, H3K4me3_peaks_Trynka; Transcription, Transcr_Hoffman.flanking.500; H3H27ac, H3K27ac_PGC2.flanking.500; Repressed, Repressed_Hoffman.

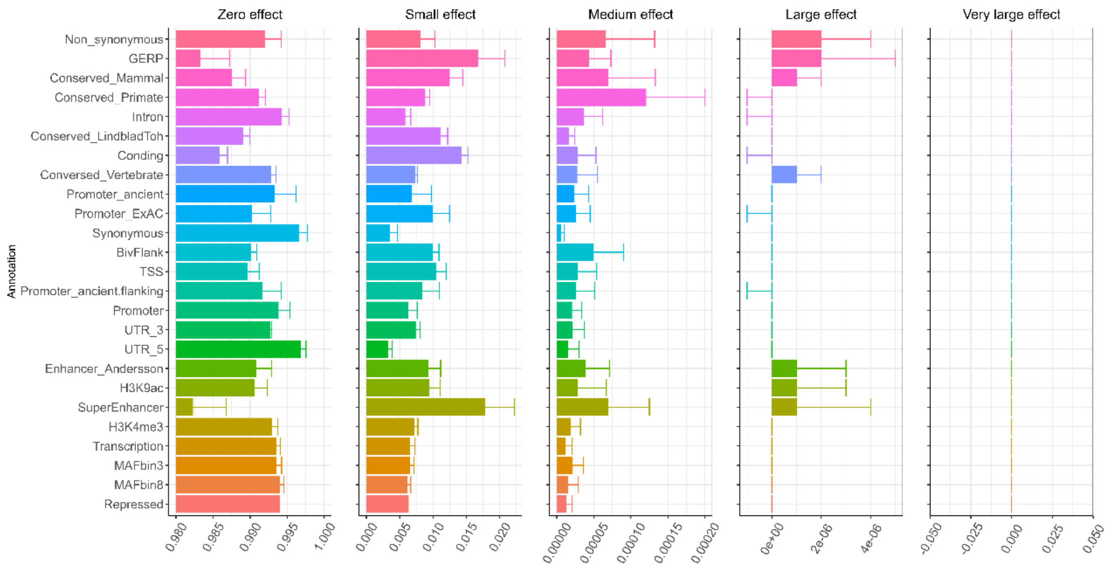

Supplementary Figure 8 Proportion of SNPs in a functional category assigned to five different mixture components based on their estimated effect sizes. We used SBayesRC to model SNP effect sizes as a mixture of five components (zero, small, medium, large and very large effect). For each functional category, the proportions of SNPs assigned to these effect size components were estimated, reflecting whether the observed enrichment in per-SNP heritability was due to a higher number of causal variants or larger effect sizes in that category. This figure shows the top 20 and bottom 5 functional categories ranked by per-SNP heritability enrichment. Functional categories correspond to annotations from the BaseLineLD model v2.2. Error bars denote the posterior standard deviation (posterior SD) obtained from the Markov Chain Monte Carlo (MCMC) samples of the SBayeRC model.

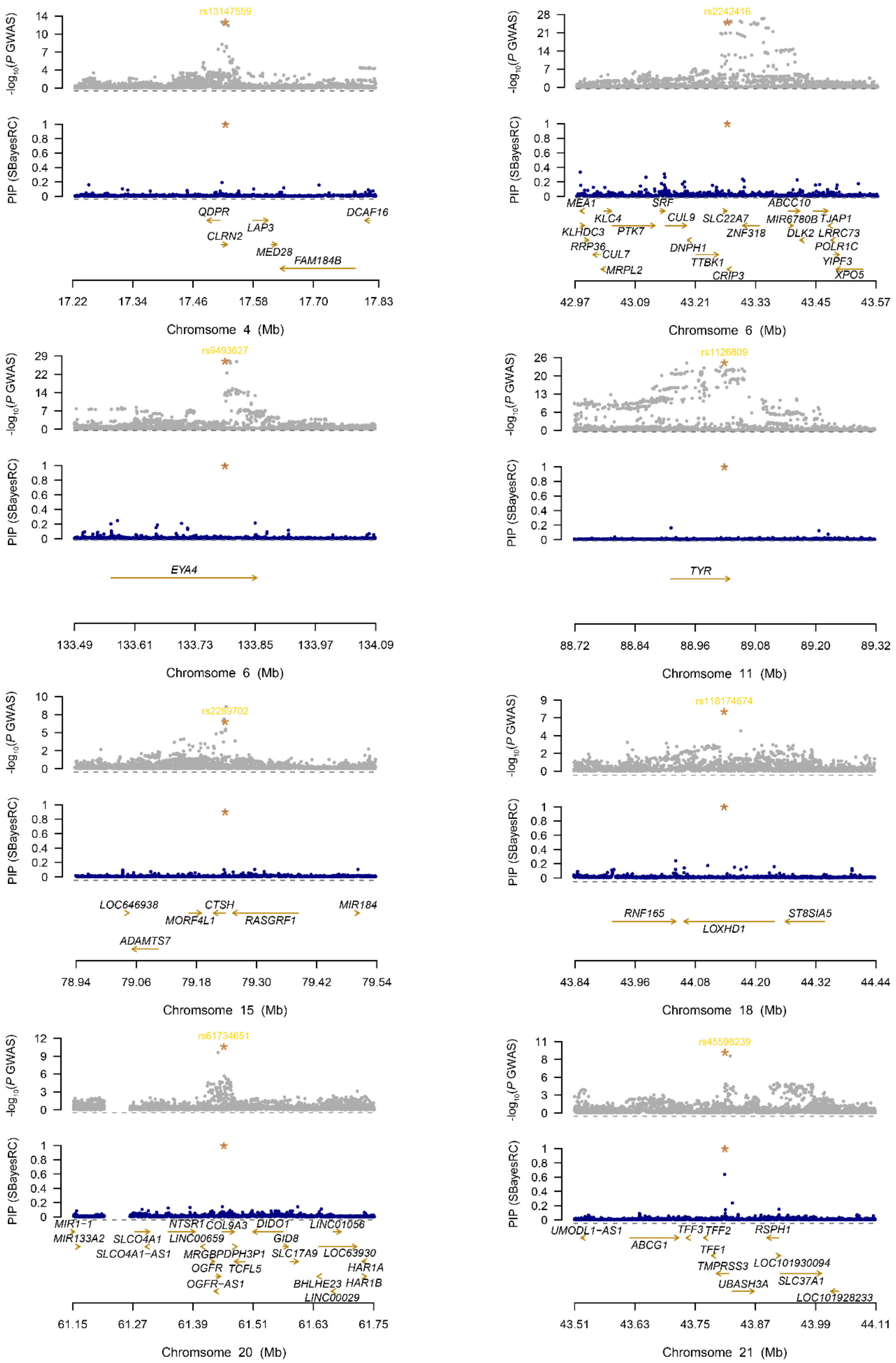

rs13147559
-log10(P GWAS)
PIP (SBayesRC)
QDPR
LAP3
CLRN2
MED28
FAM184B
DCAF16
17.22 17.34 17.46 17.58 17.70 17.83
Chromosome 4 (Mb)
rs2242416
MEA1
KLC4
KLHDC3
PTK7
RRP36
CUL7
MRPL2
SRF
CUL9
DNPH1
TTBK1
SLC22A7
ZNF318
CRIP3
ABCC10
MIR6780B
TJAP1
DLK2
LRRC73
POLR1C
YIPF3
XPO5
42.97 43.09 43.21 43.33 43.45 43.57
Chromosome 6 (Mb)
rs9493627
EYA4
133.49 133.61 133.73 133.85 133.97 134.09
Chromosome 6 (Mb)
rs1126809
TYR
88.72 88.84 88.96 89.08 89.20 89.32
Chromosome 11 (Mb)
rs2289702
LOC646938
CTSH
MORF4L1
RASGRF1
MIR184
ADAMTS7
78.94 79.06 79.18 79.30 79.42 79.54
Chromosome 15 (Mb)
rs118174674
RNF165
LOXHD1
ST8SIA5
43.84 43.96 44.08 44.20 44.32 44.44
Chromosome 18 (Mb)
rs61734651
MIR1−1
MIR133A2
SLCO4A1
SLCO4A1−AS1
NTSR1
LINC00659
MRGBP
COL9A3
DPH3P1
OGFR
TCFL5
OGFR−AS1
DIDO1
GID8
SLC17A9
BHLHE23
LINC01056
LOC63930
HAR1A
HAR1B
LINC00029
61.15 61.27 61.39 61.51 61.63 61.75
Chromosome 20 (Mb)
rs45598239
UMODL1−AS1
ABCG1
TFF3
TFF2
TFF1
TMPRSS3
UBASH3A
RSPH1
LOC101930094
SLC37A1
LOC101928233
43.51 43.63 43.75 43.87 43.99 44.11
Chromosome 21 (Mb)

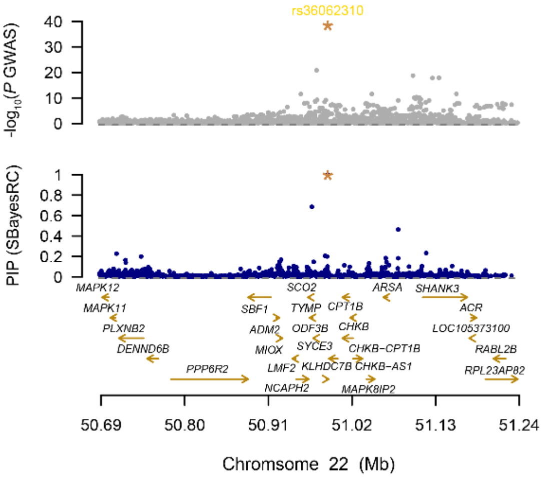

Supplementary Figure 9 Regional association plots of the 9 missense variants identified from the genome wide fine mapping (GWFM). The top track shows the GWAS *P*-value in -log10 scale, and the second track shows the similar plot but with the PIP from GWFM for ARHL.

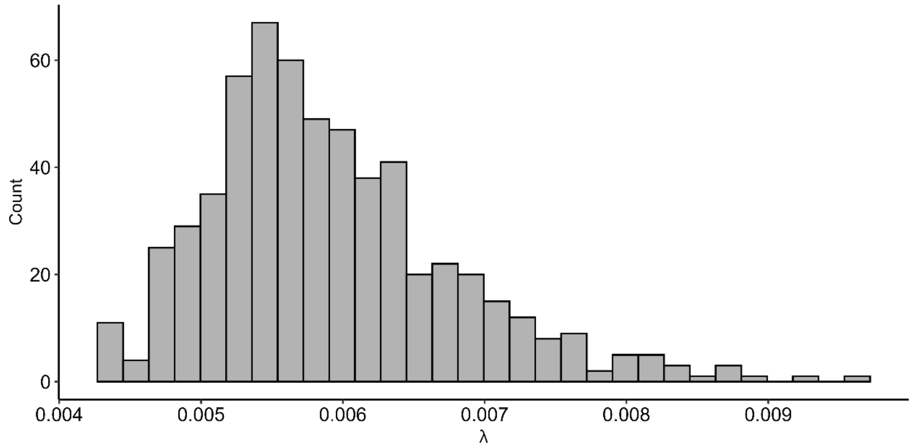

Supplementary Figure 10 Concordance between the LD reference and ARHL GWAS.

The histogram shows the distribution of λ values estimated from SuSiE diagnostics.

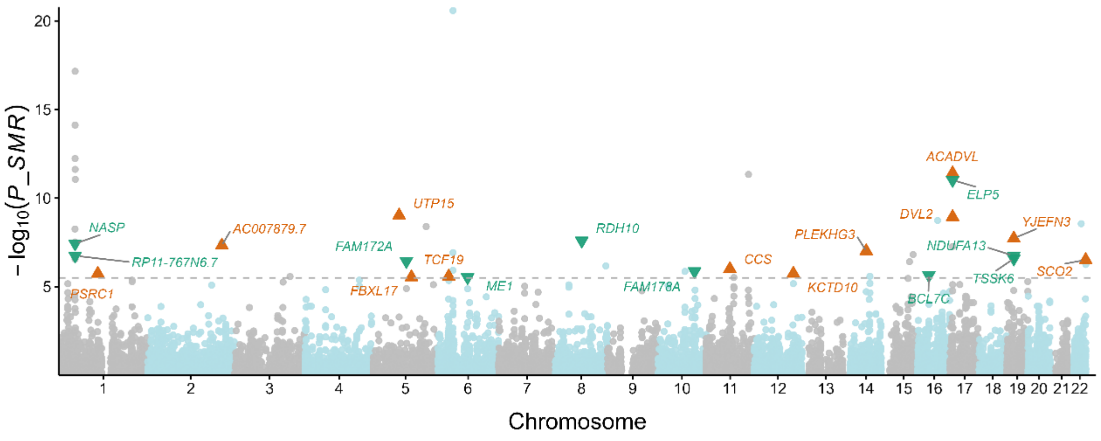

Supplementary Figure 11 Manhattan plot of SMR tests for associations between gene expression and ARHL. The grey horizontal line denotes the significance threshold after Bonferroni correction (*P_SMR* < 3.20×10^{-6}) for SMR analysis. Triangles denote significant genes that passed both the SMR and HEIDI tests (*P_HEIDI* > 0.01). Upward-pointing orange triangles indicate positive associations, while downward-pointing green triangles indicate negative associations.

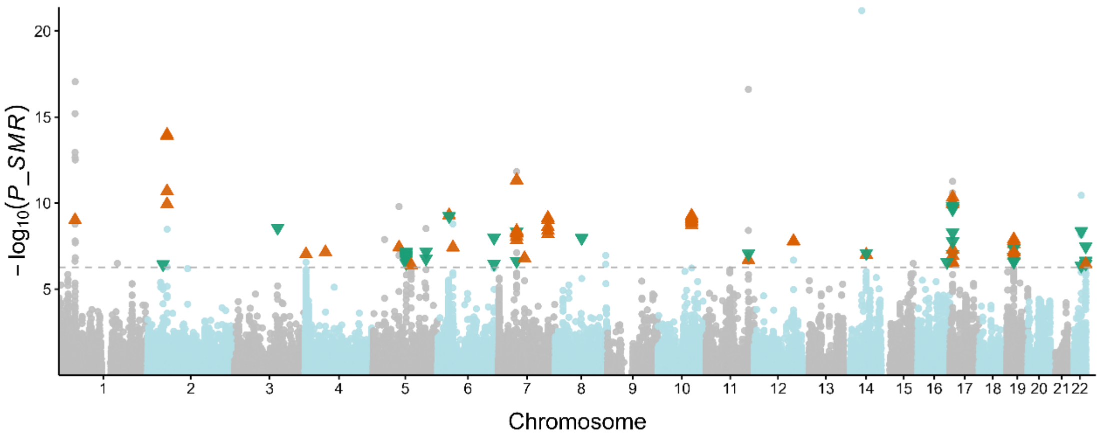

Supplementary Figure 12 Manhattan plot SMR tests for association between DNA methylation sites and ARHL. The grey horizontal line denotes the significance threshold after Bonferroni correction ($P\_SMR < 5.39\times10^{-7}$) for SMR analysis. Triangles denote significant DNA methylation sites that passed both the SMR and HEIDI tests ($P\_HEIDI > 0.01$). Upward-pointing orange triangles indicate positive associations, while downward-pointing green triangles indicate negative associations.

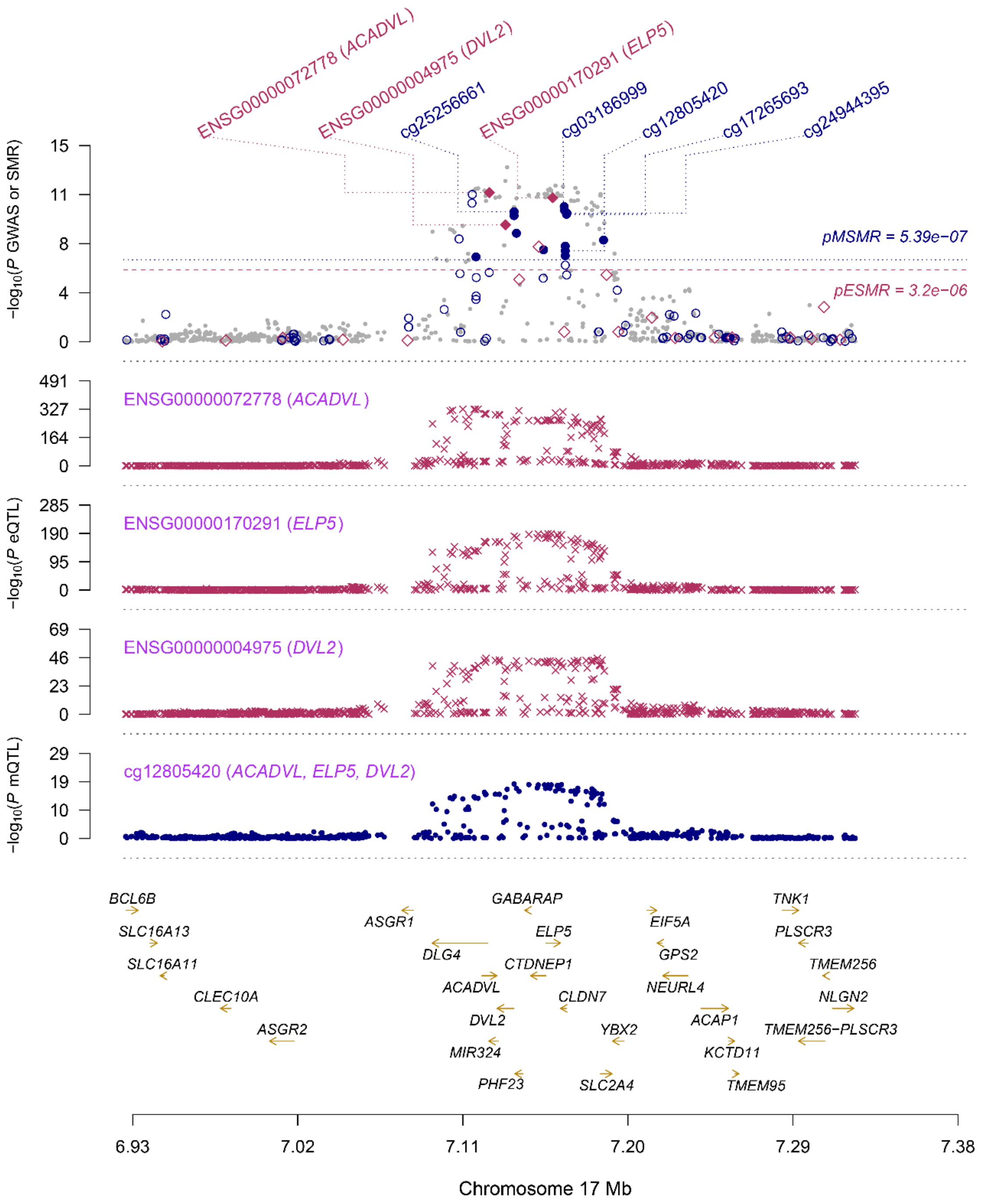

Supplementary Figure 13 Prioritizing genes and regulatory DNA methylation sites for ARHL at the 17p13.1 locus. Results of SMR analysis that integrates GWAS summary statistics with the mQTL and eQTL studies are shown. The top plot shows -log$_{10}$(P value) of SNPs from the GWAS meta-analysis for ARHL. The red diamonds and blue circles represent -log$_{10}$(P value) from the SMR tests for associations of eQTL and mQTL probes with AHRL, respectively. Solid diamonds and circles represent the probes not rejected by the HEIDI test. The second plot (red diamonds) shows -log$_{10}$(P value) of the SNP

association for eQTL probe ENSG00000072778 (tagging *ACADVL*), ENSG00000170291 (tagging *ELP5*) and ENSG00000004975 (tagging *DVL2*). The third plot (blue circles) shows -$\log_{10}$(*P* value) of the SNP association with mQTL probes cg12805420, which co-regulated three genes.

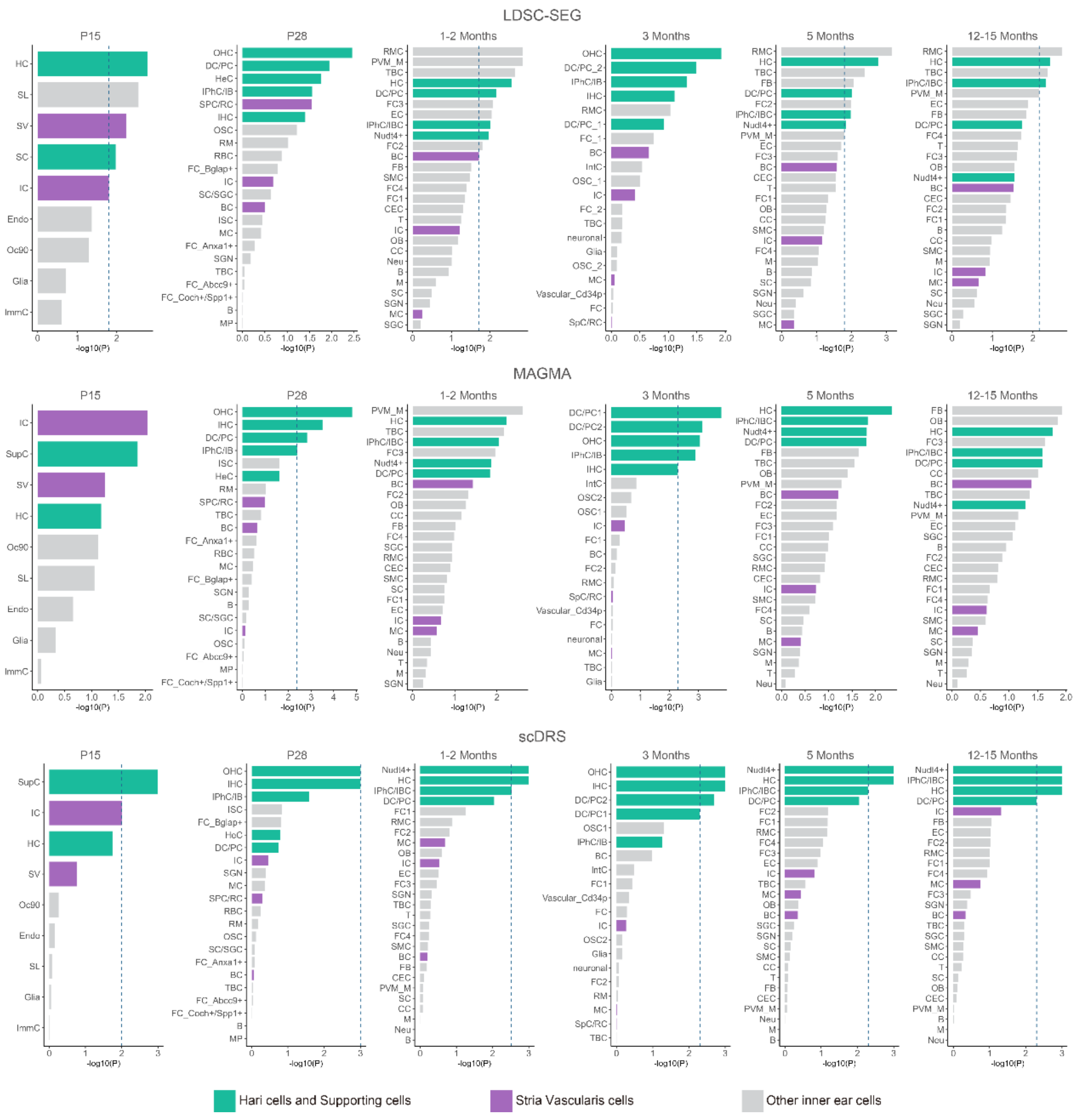

Supplementary Figure 14 Cell-type-specific heritability enrichment across three methods using comprehensive scRNA-seq datasets used by Eshel et al. Each row corresponds to one method. The first column shows results from the scRNA-seq dataset from Jean et al. (P8-20), and the remaining columns show results from the scRNA-seq dataset used by Eshel et al. Significance was controlled at FDR < 0.05 using the Benjamini-Hochberg (BH) procedure. The dashed line shows -log10(P), where P is the dataset specific nominal threshold (i.e., the raw P of the least BH-significant test). If absent, no tests passed the BH-FDR < 0.05 threshold. B, B cell; BC, basal cell; CC, chondrocyte; OB, osteoblast; CEC, capillary endothelial cell; DC/PC, Deiters cell and pillar cell; EC, epithelial cell; FB, fibroblast; FC1, fibrocyte 1; FC2, fibrocyte 2; FC3, fibrocyte 3; FC4, fibrocyte 4; HC, hair cell; HeC, Hensen's cell; IC, intermediate cell; ImmC, Immune cells; IntC, Interdental

cell; IHC, inner hair cell; IPhC/IBC, inner phalangeal cell and inner border cell; ISC, inner sulcus cell; M/MP, macrophage; MC, marginal cell; Neu, granulocyte/neutrophil; Nudt4+, Nudt4+ pillar cell; OHC, outer hair cell; OSC, outer sulcus cell; PVM_M, perivascular resident macrophage-like melanocyte; RBC, red blood cells; RMC, cells in Reissner's membrane; SC, Schwann cell; SGC, satellite glial cell; SGN, spiral ganglion neuron; SL, spiral ligament; SMC, smooth muscle cell; SpC/RC, spindle cell/root cell; SV, stria vascularis; T, T cell; TBC, tympanic border cell.

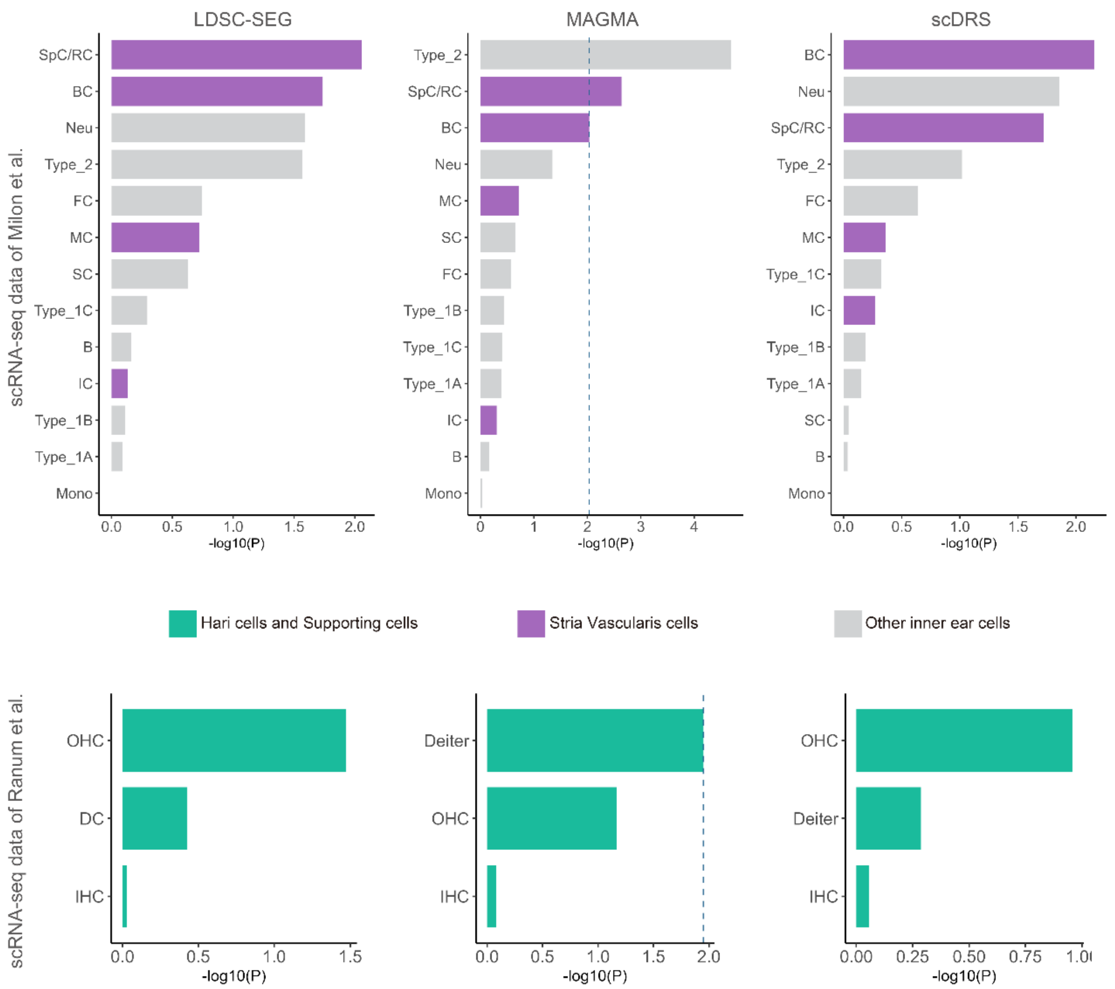

Supplementary Figure 15 Cell-type-specific heritability enrichment across three methods using scRNA-seq dataset used by Trpchevska et al. Each column corresponds to one method. The upper panel shows results based on the dataset from Milon et al. and lower panel shows results based on the dataset from Ranum et al. Significance was controlled at FDR < 0.05 using the Benjamini-Hochberg (BH) procedure. The dashed line shows -log10(P), where P is the dataset specific nominal threshold (raw P of the least BH-significant test). If absent, no tests passed the BH-FDR < 0.05 threshold. B, B cell; BC, basal cell; DC, Deiters cell; FC, fibrocyte; IC, intermediate cell; IHC, inner hair cell; MC, marginal cell; Mono, Monocyte; Neu, granulocyte/neutrophil; OHC, outer hair cell; SC, Schwann cell; SpC/RC, spindle cell/root cell.
