# Supplementary material for: Multi-ancestry GWAS of age-related hearing loss identifies 140 loci and key cellular mechanisms
Source: Nat Commun. 2026 Feb 21;17:4325. doi: 10.1038/s41467-026-69894-z (PMC13172361; doi:10.1038/s41467-026-69894-z)
Supplement: Supplementary file 3 — Description of Additional Supplementary Files [file 41467_2026_69894_MOESM3_ESM.pdf]

Description of Supplementary Data

File Name: Supplementary Data 1

Description: The summary information for 25 GWAS datasets used in this study.

File Name: Supplementary Data 2

Description: A total of 140 independent variants associated with ARHL at P < 5E-8 from the cross-ancestry meta-analysis.

File Name: Supplementary Data 3

Description: Complete genetic correlation results between ARHL and 1,738 UK Biobank traits estimated by BADGERS.

File Name: Supplementary Data 4

Description: Summary of the categories based on their clinical or functional relevance of 121 significant associations identified by BADGERS after Bonferroni correction (P < 2.88E-5).

File Name: Supplementary Data 5

Description: Genetic correlation between ARHL and other traits estimated by LDSC.

File Name: Supplementary Data 6

Description: Causal effect estimates from GSMR using ARHL as outcome.

File Name: Supplementary Data 7

Description: Causal effect estimates from multiple Mendelian Randomization methods using ARHL as outcome.

File Name: Supplementary Data 8

Description: Summary of the 165 credible set SNPs for ARHL identified from GWFM analysis.

File Name: Supplementary Data 9

Description: Summary of 22 putative causal variants for ARHL identified by GWFM.

File Name: Supplementary Data 10

Description: Summary of 9 potentially causal missense variants for ARHL prioritized by GWFM.

File Name: Supplementary Data 11

Description: SuSiE-estimated PIP values for the 22 fine-mapped variants identified by GWFM.

File Name: Supplementary Data 12

Description: ARHL-associated genes identified from the SMR analysis of the GWAS summary data from our meta-analysis and the eQTL data from the eQTLGene consortium.

File Name: Supplementary Data 13

Description: ARHL-associated CpG methylation sites from the SMR analysis of the GWAS summary data from our meta-analysis and the mQTL data from McRae et al.

File Name: Supplementary Data 14

Description: Mapping the ARHL-associated CpG methylation sites to the ARHL-associated genes by the SMR analysis of the eQTL data from the eQTLGen consortium and the mQTL data from McRae et al.

File Name: Supplementary Data 15

Description: The summary information of mouse embryo at E16.5 E2S11 from MOSTA database.

File Name: Supplementary Data 16

Description: The Cauchy combination *P* value for spatial heritability enrichment of ARHL from gsMap.

File Name: Supplementary Data 17

Description: Cell-type-specific heritability enrichment across three methods using scRNA-seq dataset from Jean et al.

File Name: Supplementary Data 18

Description: Cell-type-specific heritability enrichment across three methods using scRNA-seq dataset used by Eshel et al.

File Name: Supplementary Data 19

Description: Cell-type-specific heritability enrichment across three methods using scRNA-seq dataset used by Trpchevska et al.
