# Supplementary material for: Multi-ancestry GWAS of age-related hearing loss identifies 140 loci and key cellular mechanisms
Source: Nat Commun. 2026 Feb 21;17:4325. doi: 10.1038/s41467-026-69894-z (PMC13172361; doi:10.1038/s41467-026-69894-z)
Supplement: Supplementary file 5 — Reporting Summary [file 41467_2026_69894_MOESM5_ESM.pdf]

Corresponding author(s): Yang Wu

Last updated by author(s): Nov 17, 2025

Reporting Summary

Nature Portfolio wishes to improve the reproducibility of the work that we publish. This form provides structure for consistency and transparency in reporting. For further information on Nature Portfolio policies, see our Editorial Policies and the Editorial Policy Checklist.

Statistics

For all statistical analyses, confirm that the following items are present in the figure legend, table legend, main text, or Methods section.

n/a	Confirmed	
☐	☒	The exact sample size (*n*) for each experimental group/condition, given as a discrete number and unit of measurement
☒	☐	A statement on whether measurements were taken from distinct samples or whether the same sample was measured repeatedly
☐	☒	The statistical test(s) used AND whether they are one- or two-sided<br>*Only common tests should be described solely by name; describe more complex techniques in the Methods section.*
☒	☐	A description of all covariates tested
☐	☒	A description of any assumptions or corrections, such as tests of normality and adjustment for multiple comparisons
☐	☒	A full description of the statistical parameters including central tendency (e.g. means) or other basic estimates (e.g. regression coefficient) AND variation (e.g. standard deviation) or associated estimates of uncertainty (e.g. confidence intervals)
☐	☒	For null hypothesis testing, the test statistic (e.g. *F*, *t*, *r*) with confidence intervals, effect sizes, degrees of freedom and *P* value noted<br>*Give P values as exact values whenever suitable.*
☒	☐	For Bayesian analysis, information on the choice of priors and Markov chain Monte Carlo settings
☒	☐	For hierarchical and complex designs, identification of the appropriate level for tests and full reporting of outcomes
☐	☒	Estimates of effect sizes (e.g. Cohen's *d*, Pearson's *r*), indicating how they were calculated

Our web collection on statistics for biologists contains articles on many of the points above.

Software and code

Policy information about availability of computer code

Data collection	We used existing data from the publicly available GWAS summary data of age-related hearing loss.<br>The data used for replication are sourced from the Finngen project. No software was ued for data collection in this study.
Data analysis	Our cross-ancestry GWAS meta analysis was implemented using the METAL software (the version released on 2011-03-25) with the fixed-effect inverse-variance-weighted (IVW) model. For genetic architecture analysis, we employed SBayesS to estimate joint distribution of SNP effect size and MAF. For genetic correlation analysis, we applied BADGERS to estimate trait associations by leveraging GWAS summary statistics and a reference panel of PRS weights across multiple phenotypes. We further employed GSMR (v1.1.1) method to investigate the potential causal associations between ARHL and other complex traits. Additionally, GWFM (v2.5.2) and susieR (v0.12.35) was used for fine-mapping credible set SNPs. For Summary-based Mendelian Randomization (SMR) analysis, we employed SMR software (v1.3.1) to integrate mQTL and eQTL data with cross-ancestry GWAS. Refer to the links in the main text for all analysis tools and the Methods section for details.

For manuscripts utilizing custom algorithms or software that are central to the research but not yet described in published literature, software must be made available to editors and reviewers. We strongly encourage code deposition in a community repository (e.g. GitHub). See the Nature Portfolio guidelines for submitting code & software for further information.

Data

Policy information about availability of data

All manuscripts must include a data availability statement. This statement should provide the following information, where applicable:

- Accession codes, unique identifiers, or web links for publicly available datasets
- A description of any restrictions on data availability
- For clinical datasets or third party data, please ensure that the statement adheres to our policy

All GWAS summary statistics used for ARHL meta-analysis are available as below: the ARHL GWAS of East Asian from BBJ is publicly available at https://pheweb.jp/pheno/Hearing_Loss; the ARHL GWASs of East Asian, European, African and Admixed American from MVP are available via the dbGap study accession number phs002453; the ARHL GWAS of European from Trpchevska et al. is available at https://zenodo.org/records/5769707#.Ybm6v33MKhx. The summary statistic of cross ancestry meta-analysis from this study is available at https://zenodo.org/records/17141085. The summary-level xQTL data used for SMR are available as follow: eQTL data from eQTLGen project are available at https://www.eqtlgen.org/cis-eqtls.html and the meta-analysis data of mQTL from LBC and BSGS are available at https://cnsgenomics.com/software/smr/#Download. The Roadmap Epigenomics Mapping Consortium epigenomic annotations data are available for download at http://compbio.mit.edu/roadmap. The 1000 Genome project of European reference data (phase 3) are available at https://ftp.1000genomes.ebi.ac.uk/vol1/ftp/phase3/. The spatial transcriptomics data of mouse embryos at E16.5 used for gsMap is available at https://db.cngb.org/stomics/mosta/download. The single-cell RNA-seq data of mouse cochlea for Jean et al., Iyer et al., Eshel et al., and Sun et al. is available from the dataset access via the gEAR portal (https://umgear.org/p?s=7fd80bf5, https://umgear.org/p?s=728a05e2, https://umgear.org/p?s=bb49463b, and https://umgear.org/p?s=653896d7). Source data are provided with this paper.

Research involving human participants, their data, or biological material

Policy information about studies with human participants or human data. See also policy information about sex, gender (identity/presentation), and sexual orientation and race, ethnicity and racism.

Reporting on sex and gender	We performed the analysis in the publicly available data, as most datasets lack results for sex-stratified analysis, we are unable to perform comprehensive sex-stratified meta-analyses.
Reporting on race, ethnicity, or other socially relevant groupings	We conducted the largest cross-ancestry GWAS meta-analysis of ARHL to date, incorporating data from 1,510,447 individuals (456,613 cases and 1,053,834 controls) of European, East Asian, African and Admixed American ancestry. By integrating multi-omics data, our findings deepen the understanding of the genetic architecture and regulatory mechanisms of ARHL, linking associated genetic variants to cell types and molecular phenotypes.
Population characteristics	Our cross-ancestry GWAS meta-analysis including 1,510,447 individuals from 4 ancestry populations. The population characteristics of GWAS summary data has been described in previous studies.
Recruitment	Recruitment of the samples has been described in previous studies. We do not produce new data and have cited the previous work.
Ethics oversight	Institute of Rare Diseases, West China Hospital of Sichuan University.

Note that full information on the approval of the study protocol must also be provided in the manuscript.

Field-specific reporting

Please select the one below that is the best fit for your research. If you are not sure, read the appropriate sections before making your selection.

☒ Life sciences ☐ Behavioural & social sciences ☐ Ecological, evolutionary & environmental sciences

For a reference copy of the document with all sections, see nature.com/documents/nr-reporting-summary-flat.pdf

Life sciences study design

All studies must disclose on these points even when the disclosure is negative.

Sample size	We conducted the largest cross-ancestry GWAS meta-analysis of ARHL to date, incorporating data from 1,510,447 individuals (456,613 cases and 1,053,834 controls) of European, East Asian, African and Admixed American ancestry.
Data exclusions	Duplicated SNPs and variants with inconsistent or mismatched alleles across cohort were excluded. We further filter out SNPs with a minor allele frequency (MAF) < 0.01.
Replication	We replicated the identified independent loci using an independent dataset from the Finngen project. Replication was assessed by both effect directions and correlation between replication and discovery. The majority of replicated associations showed consistent effect directions and high correlation efficient (P < 1.38E-33), supporting the robustness of our findings.
Randomization	Randomization for sample collection was not relevant to this study because we performed the analysis in the publicly available data.
Blinding	Blinding was not relevant to this study, because we performed the analysis in publicly available data. We did not use any study design that required blinding, with the details described in the Methods section.

Reporting for specific materials, systems and methods

We require information from authors about some types of materials, experimental systems and methods used in many studies. Here, indicate whether each material, system or method listed is relevant to your study. If you are not sure if a list item applies to your research, read the appropriate section before selecting a response.

Materials & experimental systems

n/a	Involved in the study
☒	☐ Antibodies
☒	☐ Eukaryotic cell lines
☒	☐ Palaeontology and archaeology
☒	☐ Animals and other organisms
☒	☐ Clinical data
☒	☐ Dual use research of concern
☒	☐ Plants

Methods

n/a	Involved in the study
☒	☐ ChIP-seq
☒	☐ Flow cytometry
☒	☐ MRI-based neuroimaging

Plants

Seed stocks	This is not relevant to our study, because our analysis was based on human data.
Novel plant genotypes	This is not relevant to our study, because our analysis was based on human data.
Authentication	N/A
